# Tracing the Effect of Functional Rehabilitation for Improving the Quality of Life of Patients with Stroke

**Published:** 2020-05

**Authors:** Petya KASNAKOVA, Snezhana DRAGUSHEVA, Stefka IVANOVA

**Affiliations:** 1. Medical College, Medical University of Plovdiv, Plovdiv, Bulgaria; 2. Faculty of Public Health, Medical University of Plovdiv, Plovdiv, Bulgaria; 3. Faculty of Pharmacy, Medical University of Sofia, Sofia, Bulgaria

## Dear Editor-in-Chief

Worldwide mortality due to stroke and heart disease will increase to 23.6 million in 2030 (1). Bulgaria has one of the highest rates of cerebrovascular disease incidence and mortality in the world. Cerebrovascular diseases are a significant socio-medical problem, which leads to high mortality and disability rates worldwide. According to the definition of the WHO stroke is a neurological deficiency of cerebrovascular origin occurring for more than 24 h or being interrupted by death of the individual within 24 h. Every year millions of people die from stroke and millions have serious neurological deficiency, which raises serious social issues related to the disability of the patients, to the patient’s family, to the medical service and generally to the re-socialization of the patients. Today one of the main aims of medical rehabilitation and ergotherapy is to improve the quality of life, which shall meet the requirements of patients with stroke. According to the definition of WHO ”quality is an approach required to ensure each patient a set of diagnostic and therapeutic activities, which will give the best possible result in terms of the patient’s health following the current state of the medical science” (2). The quality of life is one of the most relevant issues in the management of stroke. The strict adherence to the standards of physical medicine and rehabilitation during the treatment is a prerequisite for achieving higher standard of life of patients with post-stroke hemiparesis (3).

The scope of this study was to assess the role of rehabilitation for improving the functional capacity, independence in the ergotherapeutic activities and the quality of life of the patients with stroke, as well as the necessity of a lasting and adequate to the patients’ needs rehabilitation. The patients had two seven-day hospital treatment courses in the Physical Medicine and Rehabilitation Clinics at the General Hospital “St. Panteleymon” Plovdiv, Republic of Bulgaria for the period between 2016 and 2017.

The prescription period for the studied patients was from one to ten months. Forty-three patients had two full treatment courses as between the treatment stages 4 patients dropped out because of the neurological complications that occurred. It was of great interest to us to trace the functional recovery of patients with relatively the same localization of the brain damage, which was in correlation with the rehabilitation potential and the complex rehabilitation programme.

In the acute stage of the disease, the physical therapy and rehabilitation are targeted at preventing complications in the locomotor system (contractures, decubitus), of the nervous and respiratory system (4). As a result, physical therapy is of crucial importance for the optimal physical recovery of the patients, for their re-adaptation and re-socialization. The leading role in this process is for the kinesiotherapy. It is performed by specialized methods of Kabat, Knott and Voss (5). Patient functional status is analyzed often by different methods: Brunnstrom stages of stroke recovery (6). From 68 patients with stroke with severe or moderate degree of impairment were chosen 47: 18 women (38.29 % ± 11.46 %) at average age of 63 and 29 men (61.71 % ± 9/03) at average age of 65. The other 21 patients had slight stroke incidence without paretic change and were not object of this study as the disability in these cases is not high. All participants in the study or their relatives on their behalf agreed in written to participate in the study and were informed about the rehabilitation and physical methods of treatment. The studied group was treated following the basic therapeutic program, which corresponds to the medical standards for quality health care and includes. Preformed physical factors, low-frequency pulse magnetic field, electrostimulation, laser treatment and laser puncture were the main approaches applied. In cases of serious spasticity, we applied exogenous heat – Solex, infrared (4).

The choice of specific kinesitherapeutical techniques (individual active and passive gymnastics, learning basic daily routines, ergotherapeutic activities, training in walking, exercises for stabilization of balance and walking), was done individually for each patient after precise kinesic analysis of the spasticity syndrome and functional assessment in the beginning and the end of each rehabilitation programme. An individual complex rehabilitation algorithm was used, which includes kinesitherapy and therapeutical massage: inhabiting positions under the Bobath approach for suppression of the spastically increased muscle tonus; exercises to decrease the muscle imbalance; stretching exercises; suspension of upper and lower limbs; methods for proprioceptive neuromuscular facilitation in accordance with the Kabat, Knott and Voss approach for neuromuscular stimulation; diagonal-spiral patterns for upper and lower libs of Kabat; exercises for coordination and balance (5).

The applied complex functional rehabilitation, combined with personal health care led to significantly faster reduction of the functional dysfunctions and improvement of the independence and the quality of life of the patients. However, the recovery is not sufficient in terms of the independence of the general movements and social re-integration. The patients and their relatives must be trained to apply continuously the rehabilitation activities along with the ergotherapeutical at home. The tolerant attitude on behalf of close people and relatives is essential for people with stroke after the incident. The significance of training in the complex approach is also very important for the improvement of recovery skills and the functional condition which leads to improvement of quality of life in patients with stroke.
